# Evaluation of Substance P as a New Stress Parameter in Horses in a Stress Model Involving Four Different Stress Levels

**DOI:** 10.3390/ani13071142

**Published:** 2023-03-24

**Authors:** Dominik Scholler, Yury Zablotski, Anna May

**Affiliations:** 1 Equine Hospital, Ludwig Maximilians University, 85764 Oberschleissheim, Germany; 2 Clinic for Ruminants with Ambulatory and Herd Health Services, Centre for Clinical Veterinary Medicine, Ludwig Maximilians University, 85764 Oberschleissheim, Germany

**Keywords:** noseband, equine, stress, cortisol, substance P, ridden horse ethogram, overground endoscope

## Abstract

**Simple Summary:**

The public perception of animal welfare in equestrian sports depends on factors associated with training methods and presentation of horses at equestrian events. Regarding this, correctly buckled nosebands have played an important role in recent years. Furthermore, methods were required to objectify stress and anxiety reactions in horses. Besides cortisol measurements, ridden horse ethograms were developed to quantify adverse reactions and pain-related behavior in the ridden horse. The main aim of the study was to evaluate substance P (SP) as a new stress parameter mainly addressing emotional stress reactions. Other objectives were to establish reference values for SP in horses and to compare concentrations to the established stress parameter cortisol. A ridden horse ethogram was used to simultaneously evaluate obvious adverse reactions. Plasma concentrations of SP showed large interindividual variations, whereas they stayed within more narrow ranges in individual horses. While cortisol showed a linear increase with the four applied stress levels (level 1: horses ridden with loose noseband, level 2: tight noseband, level 3: loose noseband and overground endoscope, level 4: tight noseband and overground endoscope), SP showed no correlation and may therefore be not suitable to quantify emotional stress in horses in the present minor stress model.

**Abstract:**

Stress has a significant impact on equine welfare. There are some studies on the stress response in horses ridden with tight nosebands, but little is known about other stress parameters than cortisol, which potentially could address an emotional component. In this study, blood samples of a total of 74 warmblood horses were used to establish reference values for plasma substance P (SP) concentrations. Moreover, 16 of these warmblood horses were included in a stress model. Four different stress levels (level 1: horses ridden with loose noseband, level 2: tight noseband, level 3: loose noseband and overground endoscope, level 4: tight noseband and overground endoscope) were applied to evaluate SP as a potential stress parameter in horses. Blood samples were taken at rest (t0) and directly after inducing stress (noseband tightening, insertion of endoscope; t1), as well as after 20 min of riding at all gaits (t2). A ridden horse ethogram was applied and showed that horses in the tight noseband group resorted to other stress-related behavioral issues than horses with loose nosebands. Serum cortisol showed a linear increase concurrent with the increase in stress levels with a significant difference between level 1 and level 4 (*p* = 0.043), proving that stress factors were adequate to evaluate the stress response, whereas SP did not show a correlation with the stress levels. Furthermore, concentrations of SP differed widely between horses but stayed within more narrow limits in the individual horse. As a conclusion, SP might not be a reliable stress parameter in horses in the applied minor stress model.

## 1. Introduction

In the past years, equestrian sport has repeatedly received public criticism due to controversial training methods [1]. In this context, the often very tightly buckled nosebands, which are intended to prevent the horse from opening its mouth in response to a hard hand impact, as well as from laying the tongue over the bit or sticking it out to the side, also attracted a lot of attention [2,3]. Since the horse is thus deprived of any withdrawal from a hard rider’s hand, it is a frequent point of criticism and does not correspond to the guidelines of the equestrian sport associations. The guidelines of the German Equestrian Federation (Fédération Equestre Nationale; FN) recommend that two fingers of an adult should be placed between the noseband and the bridge of the nose of the horse. However, this is not always met in practice [4,5]. Various studies have evaluated the impact of tight nosebands on so-called stress parameters, such as cortisol, heart rate variability and eye temperature measured by infrared thermography, as well as horses’ behavior [4,6,7]. In these studies, it was evident that a very tight noseband caused physiological stress responses due to its restraining nature and inhibited the expression of oral behaviors (swallowing, licking), which were increased after removal of the noseband [6]. 

Several studies also examined the connections between different training methods, head positions, fastening of the noseband and use of auxiliary reins with stress and anxiety behavior in horses by measuring cortisol, eye and facial skin temperature or heart rate variability [4,6,8,9,10,11,12,13]. In this context, a ridden horse ethogram (RHE) was developed which aimed to evaluate stressful and painful conditions in ridden horses by interpreting their facial expressions and body language [14,15]. 

Furthermore, for making stress situations in horses more objectifiable on a biochemical level, a call for suitable parameters has been made. Cortisol is the main stress parameter known to increase after stressful events [16], but little is known about its association with emotional stress [17]. In this study, the usability of substance P (SP) as a stress parameter in horses was evaluated. The neuropeptide SP was shown to be a good stress parameter in human medicine, indicating predominantly emotional- and anxiety-related stress. SP has been connected to a variety of pathophysiological processes including stress regulation and affective- and anxiety-related behavior [18]. SP is a neuropeptide composed of eleven amino acids. It is localized both inside and outside the central nervous system and plays an important role in the control of neuronal activity, secretory processes, smooth muscle activity and the regulation of blood pressure [19]. Its predominant location in the central nervous system, the amygdala [20], plays an important role in the processing of fear, anxiety and other emotions [18,21]. SP and its preferred neurokinin 1 receptor (NK1-R) have been found in brain areas that are known to be involved in stress and anxiety responses. SP is predominantly found in primary afferent neurons and is mainly produced by sensory neurons with unmyelinated fibers, which is why it is thought to play a role in pain perception [22]. Furthermore, SP is also formed by immune cells and covers a pro-inflammatory role [23]. SP can act both in a regulatory and a modulating way. The regulatory function seems to be closely linked to endogenous opioids and catecholamines and is therefore closely related to parameters associated with pain and stress [19]. As a result of exposition to stressful stimuli, a change in SP brain tissue content has repeatedly been shown. In human medicine SP was demonstrated to increase, especially with emotional stressors, and this effect depended on the severity of the stressor [18]. This reaction was also assessed in rats and showed similar results [21]. Evidence exists regarding anxiolytic effects when neurokinin 1 receptors were blocked, and SP could not exert its functions [24]. Due to its role in stress and anxiety modulation, SP and its receptor have also been discussed in the treatment of anxiety disorders and depression [21]. SP seems to exert an anti-stress effect by modulation of both biosynthesis and release of catecholamines in the adrenals, which maintain homeostasis in the catecholamine system [25]. Furthermore, SP and cortisol are unquestionably related in the stress response, because the release of SP in response to a stressor activates the hypothalamic–pituitary–adrenal (HPA) axis and triggers anxiogenic effects that result in the release of cortisol from the adrenal glands [26]. Other data show that SP does not inhibit the initial magnitude of the HPA axis response to stress but ameliorates the effect through neurokinin-1 receptors at a central level to reduce the duration of the stress response. This may be of importance in the control of transition between acute and chronic stress [27].

SP has been investigated in several studies among different species. In humans for example, the role of SP in the immune response [28,29], in wound healing [30] or its association with cardiovascular disease [31] has been described. In veterinary medicine, SP as a biomarker for objectifying pain as a stressor has so far been investigated primarily in cattle and has been identified as a suitable parameter in various studies [32,33,34,35]. In a study with calves, which showed a significant increase in the plasma concentration of SP to an average of 506.43 +/− 38.11 pg/mL [35] after castration, SP was identified as one of the most specific biomarkers for pain in cattle [34]. Studies of the stress and pain response in dairy cows with lameness [34] or clinical metritis [33] also showed a significant increase in SP in affected animals. The maximum SP plasma concentrations were 2.20 +/− 0.47 ng/mL [34] and 47.15 +/− 5.38 ng/mL [33]. The clearly heterogeneous and different values of the concentrations in different studies have already been pointed out [32]. Other studies focused on SP as a marker in feline defensive rage behavior [36]. In rodents, increases in SP in the medial amygdala resulted in anxiety-related behavior [37].

There are different studies in horses that have dealt with SP, including as a neurotransmitter in the intestine [38] or as an agent involved in the synovia in joint diseases [39,40]. One study evaluated SP and other stress markers in Tennessee Walking Horses wearing wedge pads and chains on their forefeet for five days and found no increase in concentration [41]. To date, however, there have been only limited studies on SP as a marker in connection with pain or stress in horses. 

The present study intended to collect further objective data on the usability of SP as a possible emotional stress parameter in horses by using a four-level stress model. Cortisol regarded as one of the gold standards of stress evaluation in horses was determined in serum. The ridden horse ethogram (RHE) invented by Dyson et al. (2018), which comprises 24 equine behaviors displayed by horses showing musculoskeletal pain and discomfort was also applied [28]. A ridden horse ethogram score ≥8/24 indicates pain and discomfort. This study’s design intended to evaluate the differences in the RHE in the same horses ridden four times in different settings.

For the stress model, insertion of an overground endoscope was used in addition to the described tight nosebands. Overground endoscopy is an advanced imaging technique to diagnose airway problems in exercising horses by making evaluation of upper airways possible during riding. The overground endoscope is placed in the nostril and secured to the head with a dedicated bridle. The system accompanying the endoscope is mounted underneath the horses’ saddle. Overground endoscopy is particularly useful in horses with upper respiratory noise and has been established as a valuable diagnostic method [42]. Most horses tolerate the procedure very well, but some may show signs of discomfort. Therefore, it was hypothesized that riding with an inserted overground endoscope would trigger a stress response in the horses. 

The main aims of the study were to assess the effects of a four-level stress model on established stress parameters such as cortisol and the ridden horse ethogram to verify the stress model, and to compare them with the new parameter SP to assess its context with emotional stress in horses.

## 2. Materials and Methods

### 2.1. Animals

In this study, for the stress model an experimental group of 16 warmblood horses, consisting of 12 mares and 4 geldings with an average age of 11.63 ± 3.53 years, was used. The horses were part of the teaching horses’ group of Bavaria’s Main State Stud farm “Haupt- und Landgestuet Schwaiganger” in Ohlstadt, Germany and were all kept under the same husbandry conditions. Every horse was subjected to a detailed clinical examination and only healthy animals without signs of pain, stress or lameness were used for this study. The control group consisted of 10 horses, 6 mares and 4 geldings with an average age of 13.7 ± 5.68 years, in which blood samples were taken three times according to the sampling of the study group. 

Additionally, for establishing reference SP values 48 warmblood horses of any age (12.4 ± 4.6 years), and sex (29 mares, 16 geldings, 3 stallions) were sampled once as well as the 16 experimental group horses and the 10 control group horses (a total of 74 horses). 

The animal study protocol was approved by the government of Bavaria (Oberbayern), Munich, Germany (approval AZ ROB-55.2-2532.Vet_02-21-100, February 2022).

### 2.2. Sampling Methods

Blood samples were taken via jugular venipuncture (20 mL) using 20 G needles (0.9 *×* 40 mm). For cortisol, the blood was immediately transferred into serum tubes after venipuncture and centrifuged (2000× *g* for 10 min) after 20 min. For SP vacutainers with aprotinin (BD vacutainer blood collection tubes, Becton, Dickinson and Company, Franklin Lakes, NJ, USA) were used, and the blood was centrifuged at 3500× *g* for 15 min immediately after sampling. Serum and aprotinin plasma were harvested after centrifugation, transferred into 1.5 mL Eppendorf tubes, and stored on dry ice on site until being finally stored at −20 °C until analysis. Blood samples were taken at three different time points. The zero samples (t0) for cortisol and SP were taken in the stables in the morning of each day before further handling of the horses. The second blood sample (t1) for SP was then taken in the riding arena before riding, tightening the nosebands or administering the overground endoscope. The third blood sample for cortisol and SP (t2) was taken after stress induction when riding was finished after 20 min.

Sampling in the control group was overall performed analogical to the treatment group, meaning three time intervals t0–t2, each one hour apart, were used. Blood samples were taken at rest in the morning in the stables and processing of the samples was performed as described above. The reference group was sampled once in the morning at rest in the stable.

### 2.3. Experimental Procedure

**Figure 1:**
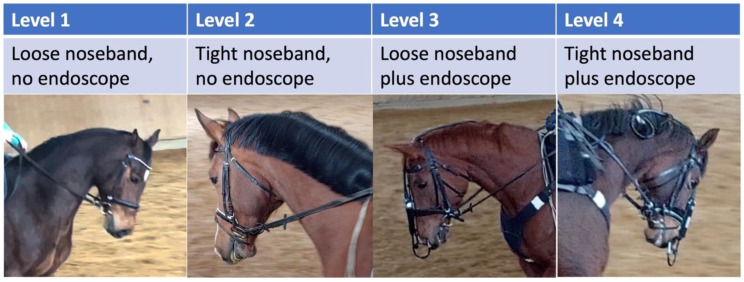
The four levels of stress induction used in this study.

In the stress model for comparison of SP, cortisol and RHE, each horse was ridden a total of four times on four different days within one week. Overall, there were four types of riding the horses which represent the four stress levels (Figure 1). Level 1 was defined by riding with a loose noseband and no endoscope, level 2 by a tight noseband and no endoscope, level 3 included riding with a loose noseband and inserted endoscope and level 4 with a tight noseband and endoscope in place. Every single horse was ridden in all the four stress levels only once. Riding was performed by a total of two professional riders with similar body conditions and riding style. Care was taken that the horses were ridden by the same rider during the experiment over the different days as the riders had to give an assessment of rideability, suppleness, willingness to perform of the horse and general riding feeling after every ride.

As described above, the sampling of the t0 blood was performed in the morning of each day in the stables, while the horses were at rest and relaxed. After sampling was completed, the horses were successively prepared for riding and led to the riding arena. When riding with overground endoscope, the horses were prepared with the necessary equipment in the stables, apart from administering the endoscope. The t1 blood samples were taken in the riding arena following administering the overground endoscope, and/or tightening the noseband or immediately before starting of riding when ridden without endoscope and with loose noseband. When ridden with loose nosebands, compliance with the two-finger rule was ensured, meaning that there was a minimum of space between the bridge of the nose and the noseband that two fingers could easily be placed in between them. When ridden with tight nosebands, the nosebands were tightened so much that there was no space between the bridge of the nose and the noseband left. Every horse was ridden in each riding type for 20 min at all gaits. While riding, videos of overground endoscopy and videos for RHE analysis were taken. After riding was finished, the t2 samples were obtained and the horses were brought back into their stables.

For establishing SP reference values, blood samples were taken from a total of 74 healthy adult warmblood horses at their stables at rest.

### 2.4. Ridden Horse Analysis 

RHE scores were analyzed using the RHE scoring system as described by Dyson et al. (2018) [14]. Every horse was filmed while riding and the videos were analyzed afterwards independently by two equine medicine specialists. The RHE score can range from 0–24, where a score of ≥8 is likely to indicate musculoskeletal pain [14]. Apart from musculoskeletal pain, stress and other suboptimal welfare conditions can result in the same behaviors listed in the RHE [43]. 

### 2.5. Analysis of Cortisol and Substance P

Substance P was measured using an SP ELISA kit (colorimetric competitive immunoassay kit; Enzo Life Sciences Inc., Farmingdale, NY, USA) with a sensitivity of 38.48 pg/mL that has been validated in bovine species [32,44] and previously been used in equines [41]. Additionally, according to manufacturer’s information, this assay is referred to as species independent for detection of SP in various body fluids. The ELISA was performed according to the manufacturer’s instructions and as described by Coetzee et al. (2008). A total of 50 μL of assay buffer was added to the nonspecific-binding and zero-standard wells, in duplicate. After that, 50 μL of the appropriate standard diluent and 50 µL of the sample were added to the appropriate wells. Thereafter, 50 μL of assay buffer was added to the nonspecific-binding wells. Following that, each well except for the total activity (TA) and blank wells received 50 μL of conjugate (alkaline phosphatase conjugated with SP) followed by 50 μL of antibody (rabbit polyclonal antibody to SP). Plates were then incubated at 22 °C on a plate shaker for 2 h at approximately 500 revolutions/min. Following incubation, contents of each plate were discarded, and wells were washed 3 times (400 μL of wash solution for each wash). After washing, wells were emptied, and plates were tapped on a paper towel to remove remaining wash buffer. Then, 5 µL of conjugate was added to the TA wells and 200 µL p-nitrophenyl phosphate substrate solution (200 μL) was added to each well, which was followed by incubation for 1 h without shaking. Finally, 50 μL of stop solution (trisodium phosphate in water) was added to each well. Immediately after the addition of the stop solution, the optical density was measured spectrophotometrically at 405 nm using a microtiter plate reader [35].

Cortisol was measured using a cortisol ELISA kit (DRG International Inc., Springfield, NJ, USA), that has been validated in horses [45,46]. The ELISA was performed as described by Bennett-Wimbush et al. (2020). During the first incubation, sample cortisol competed with cortisol conjugated to horseradish peroxidase for binding to the specific sites of the cortisol antiserum-coated wells. Following incubation all unbound material was removed by aspiration and washing. The substrate solution of tetramethylbenzidine was added, incubated for 15 min and the reaction was stopped by adding 0.5 M sulfuric acid. The optical density was read spectrophotometrically at 450 nm using a microtiter plate reader [45].

### 2.6. Establishing Reference Values

For establishing reference values, in total 74 horses were used. They consisted of 48 horses that were chosen randomly, the 10 horses of the control group and the values t0 (taken before stressing the horses in the stable facilities) of the 16 horses subjected to the different stress levels as they were taken under similar conditions.

### 2.7. Statistical Analysis

The normality of data and model residuals were tested with the Shapiro–Wilk normality test. The heteroskedasticity of model residuals was tested via the Breusch–Pagan test. The homogeneity of variances among groups was tested with Bartlett’s test. Following assumptions checks, the comparison between the four stress levels in the study group and most of the stress parameters was then performed using median-based quantile regressions, as most of the data contained contaminations, e.g., was not distributed normally. The evaluation of the ridden horse ethogram score and the different stress levels was performed using a classical linear regression, as all assumptions were satisfied. Statistical significance was set at *p* < 0.05. Model results are presented as medians or means with 95% confidence intervals. All analyses were conducted using R Statistical software (R version 4.0.3, 2020; RStudio desktop version 1.4.1103, 2021).

## 3. Results

### 3.1. Establishing Reference Values of Substance P

When looking at the 4 SP values (t0 of four different days) of the 16 horses in the experimental group, the horses did not show large intraindividual variations (SD lower than mean value in all horses; Table 1). Conversely, there were very large interindividual variations in SP plasma concentrations when t0 samples of all horses (experimental, control, reference group) were considered, displayed by the large SD (mean 2472 pg/mL, SD 1643 pg/mL; Table 2). Two horses of the reference group showed extremely high SP values (31,337 pg/mL and 23,247 pg/mL). As these 2 horses were 3- and 4-year-old mares which were brought to a different stable the same morning, the values were not considered. Table 1 and Table 2 show mean and median values as well as the standard deviations (SD) and interquartile ranges (IQR) of the SP plasma concentrations in pg/mL.

As a result, the reference range obtained in all examined horses in this study reached from 643 pg/mL to 8019 pg/mL with large variations between individual horses.

When looking at the difference between the sexes, male individuals showed mean SP plasma concentrations of 2303 ± 1449 pg/mL, while the values of mares were 2533 ± 1706 pg/mL. Although some studies pointed out gender differences in SP in humans [47] or sex differences of the SP content of certain brain areas in rats [48], no clear conclusions regarding a gender difference in horses could be drawn in this study due to a relatively small number of samples. The gender distribution was far from even and with a share of only 36%, male horses were clearly underrepresented. As a result, no further conclusions on gender differences in SP plasma concentrations in horses could be drawn, which would require further investigation and studies. 

### 3.2. Four Different Stress Levels—Correlation with Stress Parameters

#### 3.2.1. Cortisol

**Figure 2:**
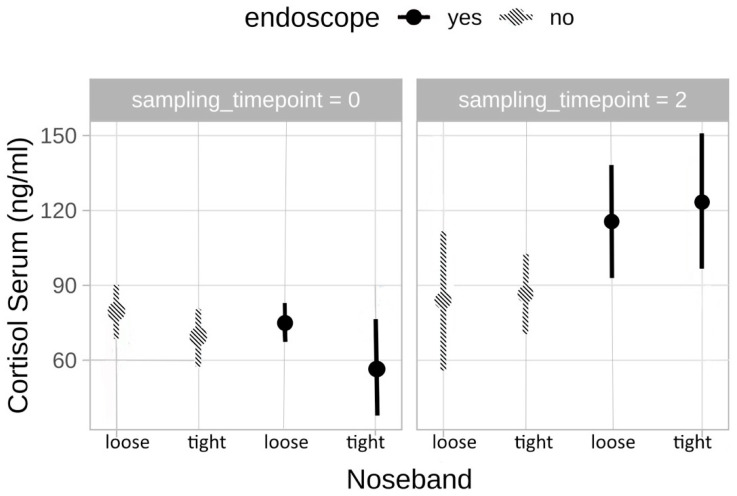
Cortisol in correlation with the different stress levels (level 1 = loose noseband, no endoscope, level 2 = tight noseband, no endoscope, level 3 = loose noseband plus endoscope, level 4 = tight noseband plus endoscope) at timepoint 0 (t0) and 2 (t2) after riding (n = 16). There was a significant increase between stress level 2 and 3 (*p* = 0.039), stress level 1 and 4 (*p* = 0.043) and as stress level 2 and 4 (*p* = 0.020).

Cortisol showed a linear increase according to the 4 stress levels with serum cortisol concentrations reaching from 56.4 ng/mL to 150.4 ng/mL when t2 values (after the horse had been stressed) were compared. The lowest overall cortisol concentrations were measured in horses at stress level 1, whereas stress level 4 displayed the highest concentrations. While the increase in cortisol was not significant in horses ridden without an endoscope (stress level 1 and 2; *p* = 0.875), there was a significant increase between horses ridden without an endoscope and those ridden with an inserted endoscope (stress level 2 and 3; *p* = 0.039). Furthermore, comparing stress level 1 with stress level 4, as well as stress level 2 and 4 showed a significant increase in cortisol concentrations (*p* = 0.043 and *p* = 0.020, respectively) (Figure 2). Within the two levels of the horses ridden with an inserted endoscope there was no significant increase (stress levels 3 and 4, *p* = 0.656). 

#### 3.2.2. Substance P

**Figure 3:**
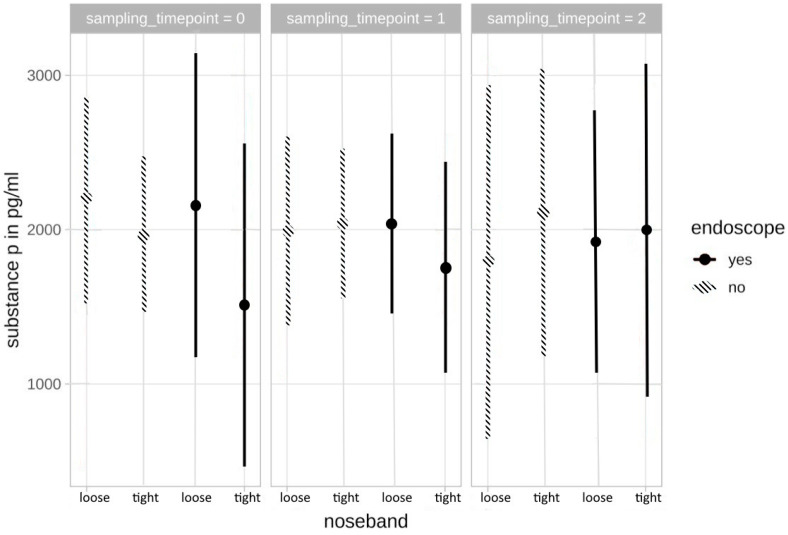
SP plasma concentrations in correlation with the different stress levels at t0–t2.

There was no significant correlation between SP plasma concentrations and the four different stress levels. SP concentrations did not allow any conclusions to be drawn with regards to the strength of the different stress levels, and no trend could be identified. Neither was there a statistically significant difference when focusing on the acute stress reaction (difference t0/t1) after the noseband was tightened or the overground endoscope was inserted (level 1: *p* = 0.923, level 2: *p* = 0.592, level 3: *p* = 0.912, level 4: *p* = 0.873, respectively), nor was there any significant difference between t0 and t2 (after riding) for any of the stress levels (level 1: *p* = 0.670, level 2: *p* = 0.950, level 3: *p* = 0.748, level 4: *p* = 0.924, respectively) (Figure 3).

### 3.3. Ridden Horse Ethogram (RHE)

**Figure 4:**
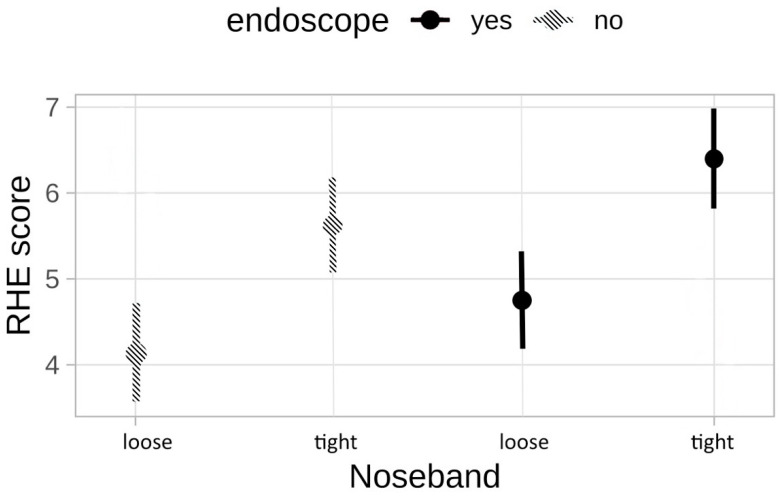
Parameters of the Ridden Horse Ethogram (RHE) shown by the horses ridden with loose or tight nosebands and with and without an inserted endoscope. There was a significant increase in the displayed stress behavior in horses ridden with tight nosebands regardless of the inserted endoscope (*p* = 0.0003).

When comparing the RHE scores between the different stress levels, a significant difference (*p* = 0.0003) was found between the horses ridden with loose and tight noseband regardless of whether the endoscope was inserted or not (Figure 4).

**Figure 5:**
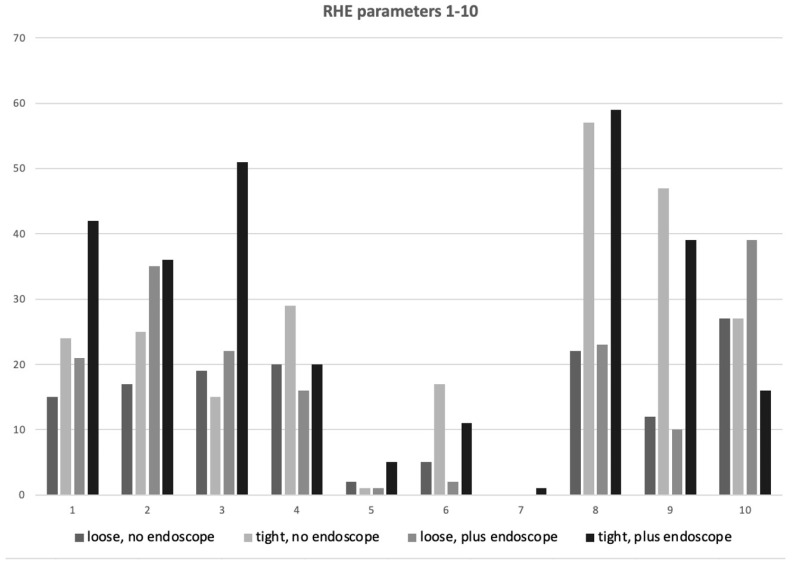
Frequency of individual RHE parameters 1–10 shown by the horses of each group (n = 16).

While the group with the loose noseband mainly showed behavioral changes such as head tilt, head in front of vertical or mouth opening (RHE number 2, 3, 10), the horses which were not able to open their mouths showed much more backwards rotated ears, exposed sclera, or intense stare (RHE parameters 6, 8, 9) (Table 3). The frequency of every displayed parameter in the separate groups is shown in Figure 5. Display of many RHE stress parameters also correlated with the riders’ impression that the horses were more dissatisfied with the tight noseband independently from the endoscope.

The RHE scores correlated neither with the cortisol concentrations (level 1: *p* = 0.967, level 2: *p* = 0.419, level 3: *p* = 0.990, level 4: *p* = 0.268), nor with the SP levels (level 1: *p* = 0.929, level 2: *p* = 0.353, level 3: *p* = 0.456, level 4: *p* = 0.625).

### 3.4. Correlation between Stress Parameters and Ridden Horse Ethogram (RHE)

#### 3.4.1. Cortisol

There was no correlation between serum cortisol concentrations and the ridden horse ethogram score when the number of parameters displayed by the horses were considered (see Section 3.3). High cortisol concentrations did not match with the highest RHE scores, and the same applies to low serum cortisol concentrations and the lowest RHE scores.

#### 3.4.2. Substance P

SP showed, just like cortisol, no correlation between the number of parameters of the RHE score displayed by the horses and changes in the plasma concentration of SP (see Section 3.3). No statistically significant correlation between high or low SP concentrations and the level of the RHE score could be pointed out.

## 4. Discussion

Because equestrian sport has increasingly come under public criticism in recent years due to its training methods, there has also been a call for parameters that make stress conditions in horses objectifiable. In this study, the usability of substance P (SP) as a stress parameter in horses was evaluated. To determine whether SP increases with stress, a four-level stressor model was used. To classify the results, SP values were compared to established stress parameters such as cortisol. For focusing on SP as a potential marker of emotional stress in horses, an RHE and the riders’ impression when riding the horses were also considered.

The present study shows that plasma concentrations of SP can regularly and reliably be measured in horses. The results for establishing reference values revealed, as seen in ruminants, a wide range of SP concentrations among individuals (see Table 1). The problem of the heterogenous results of research work and different SP concentrations among the studies has therefore been addressed in a review article [32]. According to this review, the main reasons for heterogenous outcomes concerning SP concentrations were too inconsistent study designs, cohorts and procedures [32]. Most of the studies had no control group and a lack of studies aiming for reference values in adult, healthy cattle and calves has been pointed out [32]. SP concentrations in horses and ruminants seem to be on similar levels and comparable in their heterogenous distribution width. In ruminants though, concentration values seem to differ more among different studies and study designs than within one study. In contrast, the SP plasma concentrations of horses within this study differed significantly, even when the same study protocol was used. This finding indicates even higher SP plasma concentration ranges in horses than in cattle. Apart from that, SP in individual horses shows a significantly smaller variation than in the whole cohort. This finding in the small study group evaluated in this study could indicate that SP may be a more individual parameter in horses, where changes in the SP concentration in individuals are more representative than comparing them to different horses. On the other hand, this may complicate an establishment of general reference values and should be evaluated again using a larger cohort of horses.

The stress model utilized in this study consisted of the use of a loose versus tight noseband and the additional application of an overground endoscope as it is regularly used to examine the upper airways in horses. Several studies have already shown a link between tight nosebands and the triggering of stress in horses [4,6], whereas the stress effect of applying an overground endoscope has not been looked at further in this context. Nonetheless, administering an endoscope through the nasal cavity in non-sedated horses can be assumed as a considerable stressor, not only represented by regular defensive behaviors. Additionally, riding with the administered overground endoscope is likely to cause stress and discomfort in the horses, attributed by the foreign body irritation in the nose and the pharynx. The results of this study show a linear increase in cortisol correlating with the different stress levels (lowest serum cortisol concentrations in level 1 and the highest concentrations in level 4 with significant increases between level 1 to 4, 2 to 3 and 2 to 4), which proves the triggering of a stress response and points out the suitability of the used four-level stress model. In studies where horses were exposed to very low levels of stress (lunging) no cortisol response was triggered [49].

In human medicine, the relationship between cortisol and emotional stress has been examined and differences in response to psychological stress were associated with differences in certain personality traits [50]. Although cortisol reliably rose with the stress levels, the SP concentrations, on the other hand, showed no significant correlation with the intensity of the four different stress levels. A possible reason for SP not matching with the stress model and cortisol concentrations might be due to the lack of effective or strong enough stressors used in this study [21]. In ruminants SP concentrations have mainly been investigated in more painful diseases and procedures than the minor stressors used in our model [34,35]. Although the horses were stressed, looking at the studies in ruminants, it could be stated that a much stronger stressor or a painful stimulus is needed for significant increases in SP concentrations. Hence, further investigation on SP concentrations in different stress models or diseased horses with painful conditions, for example with severe lameness or colic is needed. There have also been varying results in ruminants on the correlation between cortisol and SP. Because SP is considered a reliable parameter in cattle, electroejaculation was considered non-painful in one study since concentrations of SP remained low despite increasing cortisol levels [51].

In humans the effect of acute psychological stress on beta-endorphin and SP plasma concentrations was investigated in one study [52]. Whereas SP levels seemed to be unaffected by parachuting, beta-endorphin concentrations increased immediately after jumping [52]. A remarkable finding in this study was that high-anxiety persons showed higher total SP levels, while low-anxiety jumpers displayed lower total concentration. This fact could also be the reason for the given interindividual difference in horses and would be very interesting to investigate in a larger study population with the horses’ individual stress level considered.

The RHE was used to evaluate and quantify the horses’ stress behavior under saddle. Other studies have shown that horses ridden with a very tight noseband have limited facial signs of stress [53]. In our study, it became clear that the horses in the tight noseband groups resorted to other stress-related behavioral issues than the horses that were able to open their mouths and express their discomfort as a response to riders’ actions. The tight noseband horses predominantly showed behavioral changes which are regarded as so-called “learned helplessness”. This is an interesting fact and proved that the RHE might not only be suitable to help detect painful conditions in horses but also emotionally stressful situations. One limitation of the present study was the fact that the scoring system (RHE) only considered whether the different parameters were shown by the individual horse, but not how often or for how long the individual parameters were displayed. Some horses showed single parameters only briefly, while other horses showed them permanently or repeatedly, especially when ridden with tight nosebands. This fact is not reflected in the ridden horse ethogram used here. A recent review on the RHE looked at the fact that there is a risk when using the RHE as a standalone technique as there are many more aspects that may contribute to suboptimal welfare in the ridden horse. Another flaw is the equal weighting of the parameters without an evidence-base for that [43]. In this study, the results clearly showed the reliability in visible stress and coping mechanisms when horses were subjected to stressors such as tight nosebands or an overground endoscope. While still being a subjective tool relying on the expertise of the examiner, using the RHE in ridden horses may help to identify unfair training methods in equine sports.

Another limitation of the study could be that SP was measured from the plasma instead of, as in many other studies, from the brain tissue itself [18,21,37]. For example, release of SP was sensitive to emotional stressors in distinct regions of the amygdala, which is known to be a key area in processing of emotions, or the peri-aqueductal grey in rats [21,54]. One study in humans even evaluated SP levels in psoriatic and non-psoriatic skin and found an increased number of SP- and NK-1R-positive inflammatory cells in noninvolved psoriatic skin as a response to chronic stress [55]. Nevertheless, as studies in humans and ruminants have clearly shown a measurable increase in SP concentration, plasma was chosen to test the utility of the parameter in live horses in the context of a ridden horse’s stress detection and evaluation.

As already described above, one reason for the insufficient increase in SP concentration could be that SP rises with pain rather than stress, which causes discomfort but does not cause physical soreness. Another possible explanation for the lack of significant increase in SP levels might be the fact that most of the horses used in this study were very experienced riding lesson horses, that are used to various stressors such as a great number of consistently changing and/or unexperienced riders. Furthermore, it is not known whether these horses might be used to strictly tightened nosebands because there was no consistent evidence on how they were ridden throughout their riding lessons. So, habituation effects to moderate stressors during these riding lessons may also have resulted in coping, which may have led to ineffectively stressful conditions for significant SP value rises. Addressing that, a comparison with young, inexperienced horses in the same stress model may provide further information on the effectiveness of the performed stress model for SP as a biomarker for stress in horses.

## 5. Conclusions

As a conclusion, SP levels were not suitable to quantify emotional stress in our four-level minor stress model. Since cortisol increased analogously to the stress level, but SP did not, it makes the use of SP as a stress parameter in this context at least questionable. Further studies are needed to gain more information on SP levels depending on the stress level of the individual horse, and to evaluate the parameter in horses which are exposed to greater stress or painful conditions (e.g., colic).

## Figures and Tables

**Table 1:** SP plasma concentrations in pg/mL of the 16 horses of the experimental group at t0 of 4 different days. Individual mean and median values are shown as well as the intraindividual standard deviation (SD) and interquartile range (IQR).

Horse No.	Mean	SD	IQR	Median
1 (n = 4)	2492.1	513.4	776.5	2533.4
2 (n = 4)	2712.6	432.9	571.9	2812.1
3 (n = 4)	1232.1	550.6	774.5	1177.2
4 (n = 4)	1138.9	173.9	238.2	1103.5
5 (n = 4)	1269.7	123.7	137.3	1232.2
6 (n = 4)	2263.7	81.0	96.8	2279.3
7 (n = 4)	1046.8	86.6	101.9	1062.2
8 (n = 4)	843.8	74.5	95.9	852.1
9 (n = 4)	4786.4	470.1	620.1	4722.5
10 (n = 4)	1942.5	367.0	427.6	1948.4
11 (n = 4)	2378.9	588.2	630.6	2178.3
12 (n = 4)	1398.4	158.3	171.5	1416.8
13 (n = 4)	6132.6	1920.4	1235.7	6528.9
14 (n = 4)	4409.5	1860.0	2403.1	3973.8
15 (n = 4)	1990.6	484.7	456.0	2101.4
16 (n = 4)	4817.9	964.6	1408.9	4671.5

**Table 2:** Mean SP plasma concentrations in pg/mL at t0 of all horses used in this study (experimental group (n = 16), control group (n = 10), reference group (n = 46)).

	Mean	SD	IQR	Median
(n = 72)	2472	1643	1764	2007

**Table 3:** RHE behavior (1–10) shown by the horses in the different settings (n = 16), regardless of the frequency of the behavioral changes in the respective group.

RHE Score	RHE Description	Loose Noseband, No Endoscope	Tight Noseband, No Endoscope	Loose Noseband Plus Endoscope	Tight Noseband Plus Endoscope
1	Repeated changes of head position (up/down).	38% (6/16)	50% (8/16)	44% (7/16)	88% (14/16)
2	Head tilted/tilting repeatedly	44% (7/16)	44% (7/16)	50% (8/16)	63% (10/16)
3	Head in front of vertical (>30°) for >10 s	31% (5/16)	44% (7/16)	44% (7/16)	75% (12/16)
4	Head behind vertical (>10°) for >10 s	50% (8/16)	56% (9/16)	44% (7/16)	56% (9/16)
5	Head position changes regularly, tossed or twisted.	6% (1/16)	6% (1/16)	6% (1/16)	6% (1/16)
6	Ears rotated back behind vertical or flat >5 s.	19% (3/16)	38% (6/16)	13% (2/16)	31% (5/16)
7	Eyelids closed or half closed for 2–5 s.	0% (0/16)	0% (0/16)	0% (0/16)	6% (1/16)
8	Sclera exposed repeatedly	75% (12/16)	81% (13/16)	69% (11/16)	88% (14/16)
9	Intense stare for >5 s	50% (8/16)	81% (13/16)	44% (7/16)	75% (12/16)
10	Mouth opening, shutting repeatedly for >10 s	69% (11/16)	38% (6/16)	69% (11/16)	50% (8/16)

## Data Availability

The data presented in this study are available on request from the corresponding author.
